# Prospective Analysis of Residual Urine Volume and Its Association With Intravesical Recurrence in Patients With Non-Muscle-Invasive Bladder Cancer

**DOI:** 10.7759/cureus.84023

**Published:** 2025-05-13

**Authors:** Akihiro Maeda, Shohei Tobu, Maki Kawasaki, Hiroaki Kakinoki, Mitsuru Noguchi

**Affiliations:** 1 Department of Urology, Faculty of Medicine Saga University, Saga, JPN

**Keywords:** bladder cancer, non-muscle-invasive bladder cancer, recurrence, residual urine volume, voiding dysfunction

## Abstract

Aim

This study aimed to investigate the correlation between parameters of voiding dysfunction, including residual urine volume, and bladder cancer recurrence in patients.

Methods

A total of 68 patients (52 males and 16 females) with primary non-muscle-invasive bladder cancer (NMIBC) were prospectively analyzed. Univariate and multivariate analyses were performed with bladder cancer recurrence as the dependent variable. Explanatory variables included patient demographics, pathological findings, residual urine volume measured by ultrasound, and voiding symptoms assessed using the International Prostate Symptom Score (IPSS), the IPSS Quality of Life Index (IPSS-QOL), and the Overactive Bladder Symptom Score (OABSS).

Results

Bladder cancer recurrence was found in 11 patients (8 males and 3 females). In male patients, the median residual urine volume was significantly higher in the recurrence group (24 mL, IQR 5.5-47 mL) compared to the non-recurrence group (2 mL, IQR 0-21.5 mL; p = 0.0469). Multivariate analysis identified residual urine volume as an independent risk factor for recurrence in male patients (p = 0.0276).

Conclusion

Increased residual urine volume may be independently associated with intravesical recurrence in male patients with NMIBC. Assessing and managing voiding dysfunction may help reduce the risk of recurrence in this population.

## Introduction

Non-muscle-invasive bladder cancer (NMIBC) accounts for approximately 75% of newly diagnosed bladder cancers and is primarily treated with transurethral resection of bladder tumor (TUR-Bt) as the standard initial approach [1]. TUR-Bt is both diagnostic and therapeutic; however, achieving a complete cure with a single TUR-Bt procedure is often difficult due to the multifocal nature of the disease and limitations in visualizing flat lesions.

As a result, many patients with NMIBC require additional treatments such as repeated TUR-Bt or intravesical therapy using anticancer agents or Bacillus Calmette-Guérin (BCG). These adjuvant therapies aim to reduce the risk of recurrence and progression. Despite these interventions, it is estimated that 31% to 78% of NMIBC patients experience intravesical recurrence within five years after the initial TUR-Bt procedure [2]. Moreover, a proportion of NMIBC cases eventually progress to muscle-invasive bladder cancer (MIBC) during follow-up. MIBC often requires more invasive interventions such as radical cystectomy, which can significantly impair quality of life and may be associated with poor prognosis. Thus, managing NMIBC effectively at an early stage is essential, with particular emphasis on recurrence prevention as a major clinical challenge.

Several mechanisms have been proposed to explain intravesical recurrence after TUR-Bt. One hypothesis suggests that tumor cells dislodged during resection may remain suspended in urine and subsequently reimplant onto the bladder mucosa. Silverman et al. and others have discussed the possibility that the interaction between residual cancer cells and normal urothelium plays a role in recurrence [3]. This concept, known as "tumor cell seeding," highlights the potential impact of postoperative urinary conditions on recurrence risk.

In particular, prolonged urinary retention or increased residual urine volume may enhance contact time between exfoliated tumor cells and the bladder wall, potentially facilitating reimplantation. Although some studies have examined the oncological significance of voiding dysfunction, including bladder outlet obstruction and post-void residual volume, its relationship with intravesical recurrence remains underexplored. For example, Sazuka et al. reported that a post-void residual urine volume of more than 30 mL was an independent risk factor for recurrence in a retrospective cohort of NMIBC patients [4]. However, these studies often used retrospective designs or fixed thresholds and did not account for sex-specific differences or pharmacological confounders.

To address these gaps, we conducted a prospective study to evaluate the association between residual urine volume and intravesical recurrence in a rigorously selected NMIBC cohort. By analyzing both continuous data and clinically relevant cut-off values, our aim was to clarify whether impaired voiding function contributes to recurrence risk and to provide potential insights into recurrence prevention strategies.

## Materials and methods

Study design and study population

This study was designed as a prospective observational investigation conducted at a single institution. We enrolled a total of 68 patients, comprising 52 males and 16 females, with a median age of 74 years. All patients were newly diagnosed with primary NMIBC and received initial treatment at our department between January 2017 and June 2020. Baseline clinical data were collected prior to treatment, and all participants were followed for up to two years after TUR-Bt.

Inclusion and exclusion criteria

Patients were considered eligible for the study if they underwent TUR-Bt for the first time at our institution and had a pathological diagnosis of NMIBC, including stage pTa, carcinoma in situ, or pT1. Enrollment was limited to individuals who provided written informed consent for both participation in the study and subsequent follow-up evaluations. The inclusion criteria were set to ensure consistency in initial diagnosis and treatment exposure.

Exclusion criteria were established to eliminate confounding factors that could affect voiding function or recurrence risk. Specifically, patients were excluded if they had neurogenic bladder, pelvic organ prolapse of grade II or higher, current use of psychotropic medications, or indwelling urethral catheters. In addition, patients who underwent simultaneous transurethral resection of the prostate (TUR-P) were excluded to avoid confounding effects on voiding function and recurrence outcomes. Based on these criteria, nine patients were excluded from the original cohort prior to final analysis.

Treatment and follow-up protocol

Treatment strategies followed contemporary national guidelines for NMIBC management in Japan [5]. In cases where pathological assessment of the initial TUR-Bt revealed high-grade pTa or pT1 tumors, a second TUR was performed to confirm complete resection and staging accuracy. Postoperative maintenance intravesical therapy was administered according to risk stratification. Patients classified as intermediate risk received mitomycin C (MMC) or BCG, while those at high risk received BCG alone.

Follow-up evaluations were conducted using cystoscopy to monitor for intravesical recurrence. These assessments were scheduled at the initial postoperative visit and subsequently at three-month intervals for up to 24 months (i.e., at 3, 6, 9, 12, 15, 18, 21, and 24 months after TUR-Bt). Urine cytology was also performed at each follow-up visit as part of routine surveillance to detect non-visible recurrence. Findings from each visit were systematically recorded and used for recurrence analysis.

Clinical parameters

To assess voiding function, residual urine volume was measured using transabdominal ultrasound. These measurements were performed immediately after spontaneous voiding, both at the initial visit and at each follow-up during the cystoscopic surveillance period. For each patient, the median residual urine volume across all measurement time points during the observation period was calculated and used in the analysis. In addition, patient-reported voiding symptoms were evaluated at every follow-up visit using three standardized and validated questionnaires: the International Prostate Symptom Score (IPSS), the IPSS Quality of Life Index (IPSS-QOL), and the Overactive Bladder Symptom Score (OABSS). These tools allowed for a comprehensive assessment of LUTS and patient-perceived urinary quality of life. To maintain consistency in evaluating natural postoperative bladder function, pharmacological agents for voiding dysfunction, such as α1-blockers or 5α-reductase inhibitors, were not prescribed during the study period.

Statistical analysis

We performed both univariate and multivariate analyses to examine the relationship between clinical parameters and bladder cancer recurrence. The presence or absence of recurrence served as the dependent variable, while explanatory variables included residual urine volume, IPSS, IPSS-QOL, OABSS, patient demographics, and pathological tumor features.

Continuous variables were compared using the Mann-Whitney U test, while categorical variables were assessed with the chi-square test. For multivariate analysis, logistic regression modeling was employed to identify independent predictors of recurrence. All statistical analyses were conducted using GraphPad Prism version 9.4.0 (GraphPad Software, San Diego, CA, USA). A p-value of less than 0.05 was considered statistically significant in all tests.

## Results

Among the 68 analyzed cases, bladder cancer recurrence occurred in 11 patients (8 males and 3 females). The overall recurrence rate was 16.2% (11/68), with recurrence rates of 15.3% (8/52) in males and 18.7% (3/16) in females. The median observation period for all cases was 24 months (IQR 12-24 months).

In the analysis of all the cases, the presence or absence of residual urine was not identified as a significant factor for bladder cancer recurrence. Furthermore, variables such as sex, age, the presence of hypertension, diabetes, cerebrovascular disease, and smoking history were not significant factors for bladder cancer recurrence. These clinical background characteristics and their relationship to recurrence status are summarized in Table 1. Among the patients who experienced recurrence, urine cytology was positive in one male and one female patient. In both cases, the positive cytology findings coincided with the timing of recurrence detection by cystoscopic examination. In contrast, all patients in the non-recurrence group had negative urine cytology throughout the follow-up period.

**Table 1:** Clinical characteristics of all patients with non-muscle-invasive bladder cancer, stratified by recurrence status Data are presented as median (interquartile range) or number (percentage), as appropriate. Statistical comparisons were made between the recurrence and non-recurrence groups using the Mann-Whitney U test for continuous variables and the chi-square test for categorical variables. P-values < 0.05 were considered statistically significant.

Clinical characteristics	Overall (n = 68)	Non-recurrence (n = 57)	Recurrence (n = 11)	p-value
Median age (IQR)	74 (68-80)	76 (66-82)	74 (69-75)	0.7207
Gender				
Male	n = 52 (76%)	n = 44 (85%)	n = 8 (15%)	0.7120
Female	n =16 (26%)	n = 13 (81%)	n = 3 (19%)	
Hypertension				
Present	n = 34 (50%)	n = 32 (56%)	n = 2 (13%)	0.0785
Absent	n = 34 (50%)	n = 25 (44%)	n = 9 (87%)	
Diabetes mellitus				
Present	n = 11 (16%)	n = 11 (19%)	n = 0 (0%)	0.1896
Absent	n = 57 (84%)	n = 46 (81%)	n = 11 (100%)	
Cerebrovascular disorder				
Present	n = 6 (9%)	n = 5 (9%)	n = 1 (9%)	>0.9999
Absent	n = 62 (91%)	n = 52 (91%)	n = 10 (91%)	
Smoker				
Present	n = 40 (59%)	n = 36 (63%)	n = 4 (36%)	0.1789
Absent	n = 28 (41%)	n = 21 (37%)	n = 7 (64%)	

Next, we analyzed the impact of residual urine on bladder cancer recurrence separately for males and females. Among the three female cases with bladder cancer recurrence, none had residual urine. Detailed clinical and pathological characteristics of female patients, stratified by recurrence status, are shown in Table 2.

**Table 2:** Clinical and pathological characteristics of female patients with NMIBC, stratified by recurrence status Data are presented as median (interquartile range) or number (percentage), as appropriate. Statistical comparisons between recurrence and non-recurrence groups were performed using the Mann-Whitney U test for continuous variables and the chi-square test for categorical variables. P-values < 0.05 were considered statistically significant. BCG, Bacillus Calmette-Guérin; CIS, carcinoma in situ; IPSS, International Prostate Symptom Score; MMC, mitomycin C; NMIBC, non-muscle-invasive bladder cancer; OABSS, Overactive Bladder Symptom Score; QOL, quality of life

Female	Non-recurrence (n = 13)	Recurrence (n = 3)	p-value
Median residual urine volume, mL (IQR)	6 (0-20.5)	0 (0-5)	0.2518
IPSS (IQR)	3.5 (2.75-5.0)	3.0 (1.5-15.5)	0.9143
IPSS-QOL (IQR)	2 (1.125-3)	1 (1-4.5)	0.7495
OABSS (IQR)	3 (2-3.875)	2.5 (2-5)	>0.9999
Disease-specific characteristics			
Pathological tumor grade			
Low grade	n = 9 (69%)	n = 1 (33%)	0.5179
High grade	n = 4 (31%)	n = 2 (67%)	
Pathological tumor stage			
Ta	n = 10 (77%)	n = 1 (33%)	0.2143
T1	n = 3 (23%)	n = 2 (67%)	
Concomitant CIS			
Present	n = 1 (8%)	n = 1 (33%)	0.3500
Absent	n = 12 (92%)	n = 2 (67%)	
Postoperative maintenance intravesical therapy (MMC or BCG)			
Present	n = 4 (31%)	n = 1 (33%)	>0.9999
Absent	n = 9 (69%)	n = 2 (67%)	

In males, bladder cancer recurrence was observed in eight cases (15%), and these patients had significantly higher residual urine volumes than those without recurrence. Detailed clinical and pathological characteristics of male patients, stratified by recurrence status, are shown in Table 3.

**Table 3:** Clinical and pathological characteristics of male patients with NMIBC, stratified by recurrence status Data are expressed as median (interquartile range) or number (percentage), as appropriate. The Mann-Whitney U test was used for continuous variables, and the chi-square test was used for categorical variables to compare recurrence and non-recurrence groups. P-values < 0.05 were considered statistically significant. BCG, Bacillus Calmette-Guérin; IPSS, International Prostate Symptom Score; MMC, mitomycin C; NMIBC, non-muscle-invasive bladder cancer; OABSS, Overactive Bladder Symptom Score; QOL, quality of life

Male	Non-recurrence (n = 44)	Recurrence (n=8)	p-value
Median residual urine volume, mL (IQR)	2 (0-21.5)	24 (5.5-47)	0.0469
IPSS (IQR)	6 (4-15)	6.5 (2.5-10.75)	0.5510
IPSS-QOL (IQR)	2 (1-3)	2 (1.25-2)	0.4174
OABSS (IQR)	3 (1.5-4)	4.5 (2-5)	0.7980
Prostate volume, mL (IQR)	25 (20-34)	23 (19-29)	0.5814
Disease-specific characteristics			
Pathological tumor grade			
Low grade	n = 17 (36%)	n = 5 (63%)	0.2430
High grade	n = 30 (64%)	n = 3 (37%)	
Pathological tumor stage			
Ta	n = 21 (45%)	n = 8 (100%)	0.0368
T1	n = 15 (32%)	n = 0 (0%)	
Concomitant CIS			
Present	n = 7 (16%)	n = 0 (0%)	0.5767
Absent	n = 37 (84%)	n = 8 (100%)	
Postoperative maintenance intravesical therapy (MMC or BCG)			
Present	n = 28 (64%)	n = 1 (13%)	0.0158
Absent	n = 16 (36%)	n = 7 (87%)	

**Figure 1:**
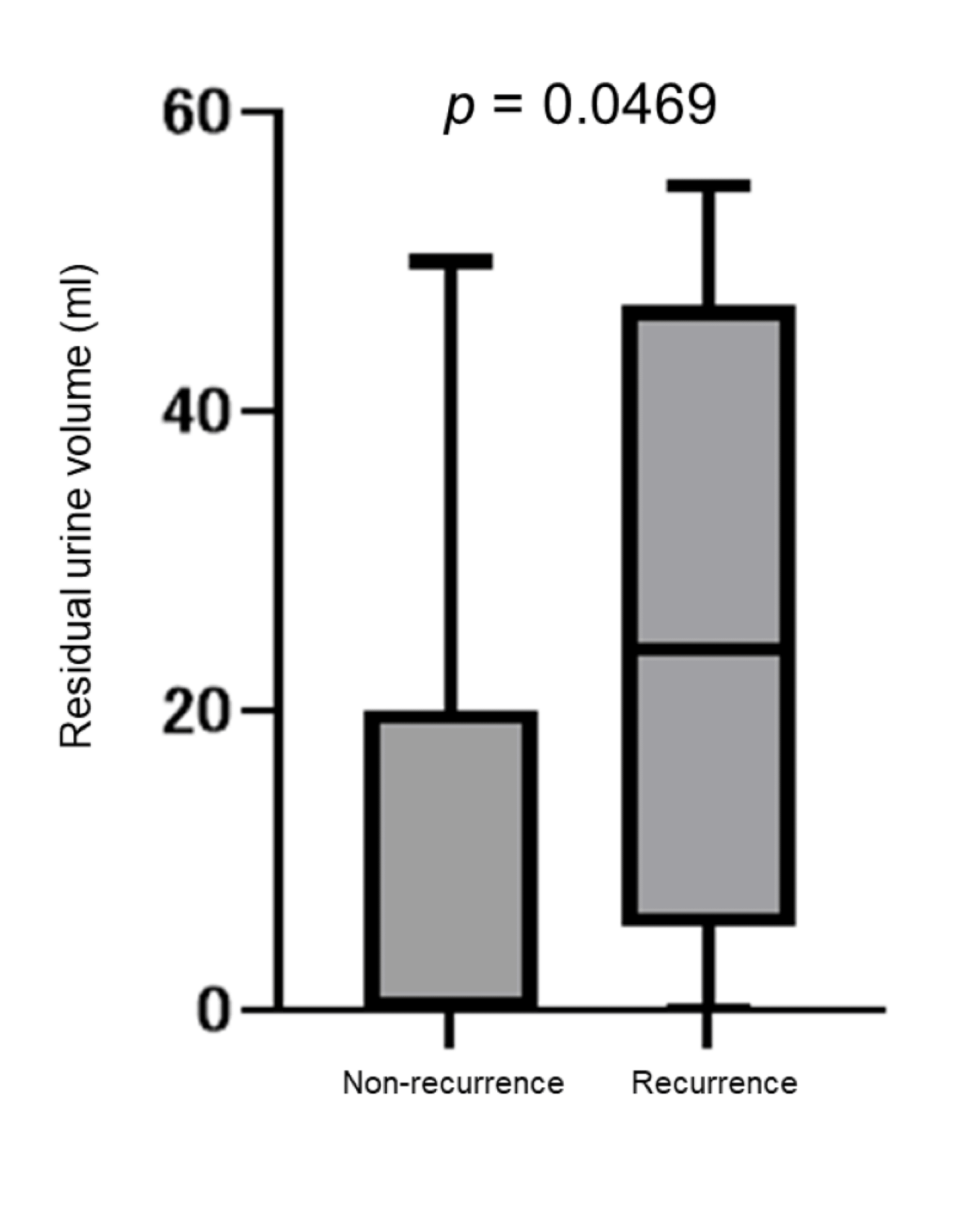
Comparison of residual urine volume between recurrence and non-recurrence groups in male patients with NMIBC Box-and-whisker plot comparing residual urine volumes in male patients with NMIBC, stratified by recurrence status. The recurrence group (n = 8) had a significantly higher residual urine volume (median 24 mL, IQR 5.5–47 mL) than the non-recurrence group (n = 44; median 2 mL, IQR 0–21.5 mL) (p = 0.0469, Mann-Whitney U test). Boxes represent the IQR, whiskers indicate the full range, and horizontal lines within the boxes denote the median values. P-values < 0.05 were considered statistically significant. IQR, interquartile range; NMIBC, non-muscle-invasive bladder cancer

In male patients, the median residual urine volume in the recurrence group (eight cases) was 24 mL (IQR 5.5-47 mL), whereas in the non-recurrence group (44 cases), it was 2 mL (IQR 0-21.5 mL), showing a significantly higher volume in the recurrence group than in the non-recurrence group (p=0.0469). This comparison is illustrated in Figure 1. Additionally, a subgroup analysis using a 30 mL cut-off for residual urine showed no statistically significant association with recurrence (p = 0.611), although none of the patients with residual urine volumes of 30 mL or more experienced recurrence. The detailed distribution of recurrence according to the 30 ml cut-off is presented in Table 4. There were no significant differences between the recurrence and non-recurrence groups in terms of the total IPSS, IPSS-QOL, OABSS total score, or prostate volume.

**Table 4:** Cross-tabulation of bladder cancer recurrence and residual urine volume using 30 mL as the cut-off in male patients Residual urine volume was categorized into two groups: less than 30 mL and 30 mL or more. The table summarizes the number of patients with and without bladder cancer recurrence in each group. Statistical significance was evaluated using the chi-square test.

Recurrence status	Non-recurrence	Recurrence	Total
Residual urine volume < 30 mL	38	8	46
Residual urine volume > 30 mL	6	0	6
Total	44	8	52

In the male patients, univariate and multivariate analyses were performed with residual urine volume, total IPSS score, IPSS-QOL, OABSS total score, prostate volume, and postoperative maintenance intravesical therapy rate as explanatory variables and the presence or absence of recurrence as the dependent variable. In the univariate analysis, residual urine volume (p=0.0167) and the rate of postoperative maintenance intravesical therapy (p=0.0245) were identified as significant factors for recurrence. However, in multivariate analysis, only residual urine volume (p=0.0276) was identified as a significant factor for recurrence. The results of these analyses are summarized in Table 5.

**Table 5:** Univariate and multivariate analyses of risk factors for bladder cancer recurrence after TUR-Bt in male patients with NMIBC Hazard ratios (HRs), 95% CIs, and p-values are shown for each variable. Univariate and multivariate logistic regression analyses were performed to assess factors associated with intravesical recurrence. Residual urine volume was treated as a continuous variable. P-values < 0.05 were considered statistically significant. BCG, Bacillus Calmette-Guérin; CI, confidence interval; HR, hazard ratio; IPSS, International Prostate Symptom Score; MMC, mitomycin C; NMIBC, non-muscle-invasive bladder cancer; OABSS, Overactive Bladder Symptom Score; QOL, quality of life; TUR-Bt, transurethral resection of bladder tumor

	Univariate analysis			Multivariate analysis		
	HR	95% CI	p-value	HR	95% CI	p-value
Residual urine volume	1.5070	1.013-1.111	0.0167	1.1270	1.039-1.319	0.0276
IPSS	0.8548	0.6706-1.013	0.1278	0.6831	0.3255-1.135	0.1893
IPSS-QOL	0.7741	0.4016-1.380	0.4069	1.2830	0.1599-7.064	0.7905
OABSS	0.7956	0.4695-1.153	0.3051	0.9224	0.1948-3.076	0.9018
Prostate volume	0.9611	0.8645-1.025	0.3538	0.9292	0.7469-1.051	0.3537
Postoperative maintenance intravesical therapy (MMC or BCG)	12.2500	1.937-239.9	0.0245	18.3500	1.309-1355	0.0718

## Discussion

This study suggests that the presence of residual urine in males may be associated with an increased risk of intravesical recurrence after TUR-Bt for NMIBC. Although this association has not been widely studied, our findings contribute to a growing body of evidence indicating the potential role of voiding dysfunction in bladder cancer prognosis.

The rate of intravesical recurrence within five years after TUR-Bt for NMIBC ranges from 31% to 78% [6]. These recurrence rates are generally understood to vary depending on factors such as the depth of invasion, malignancy grade, tumor size, and multiplicity at the time of initial diagnosis. However, there are relatively few discussions regarding voiding dysfunction or residual urine volume as significant risk factors for bladder cancer recurrence.

Sazuka et al. [4] reported that among 305 patients with NMIBC, the presence of residual urine volume >30 mL and pyuria (>10 white blood cells under 400x magnification on microscopy) were independent risk factors for bladder cancer recurrence. In addition, a report on 81 cases of total nephroureterectomy found that a residual urine volume >30 mL was significantly associated with a higher rate of intravesical recurrence [7]. Although our study did not initially adopt a fixed threshold such as 30 mL, we observed a significant association between higher residual urine volumes and recurrence risk. In a subgroup analysis using the commonly applied threshold of 30 mL for residual urine volume, no statistically significant association with bladder cancer recurrence was observed (p = 0.611). Although none of the patients in the recurrence group had residual urine volumes of 30 mL or more, the small sample size may have limited the statistical power to detect a meaningful difference. Further studies with larger cohorts are warranted to evaluate the clinical utility of this cut-off value in risk stratification.

Epidemiological studies on the relationship between lower urinary tract symptoms (LUTS) and bladder cancer have reported that 1.6% (476/30,183) of patients with LUTS were diagnosed with bladder cancer. Specifically, in cases with severe LUTS (AUA-7 score >19), the risk of bladder cancer development is 1.64 times higher than in those without LUTS (AUA-7 score <1) [8].

Although there is no consensus on the mechanism by which increased residual urine volume and voiding dysfunction elevate the risk of bladder cancer, prolonged contact between the bladder wall and urine may be a contributing factor. Silverman et al. reported that patients who urinated more than twice during the night had a decreased risk of bladder cancer [3]. Although nocturnal urination was not evaluated in our study, this prior research supports the hypothesis that reducing the contact time between urine and the bladder wall may help lower cancer risk.

Men in their 60s to 80s, the age group most affected by bladder cancer, often present with LUTS due to benign prostatic hyperplasia (BPH). Some of these patients may experience increased residual urine volume, which can result in prolonged contact between carcinogenic substances in the urine and bladder wall, potentially increasing the risk of bladder cancer development. One study has reported such a risk [9-10]. Furthermore, Matsumoto reported that injecting carcinogens into the bladder of rats with bladder outlet obstruction significantly increased the incidence of bladder cancer, suggesting that prolonged contact time between carcinogenic substances in the urine and bladder wall may contribute to the elevated risk of bladder cancer [11]. 　

The present study also suggests that an increased residual urine volume may contribute to an increased risk of bladder cancer recurrence. We believe that interventions aimed at improving voiding dysfunction could reduce contact time between the urine and the bladder wall, thereby potentially lowering the risk of recurrence. Although our cohort did not include patients who underwent simultaneous TUR-P, previous studies have suggested that combining TUR-Bt and TUR-P in patients with NMIBC and severe BPH may reduce the risk of recurrence [12-13]. In our study, while such patients were excluded to eliminate potential confounding effects on voiding parameters, the evidence from prior research indicates that concurrent TUR-P may lower the risk of intravesical recurrence in NMIBC patients with significant BPH. Further investigation is warranted to determine whether therapeutic intervention for voiding dysfunction, such as TUR-P, can effectively contribute to recurrence prevention when appropriately indicated.

Several limitations of this study should be noted. First, the sample size was relatively small, consisting of only 68 participants. Second, the follow-up period was limited to two years, which may not capture all relevant recurrence events. Third, subgroup analyses involving patients who received postoperative intravesical chemotherapy are prone to selection bias, as these individuals were primarily classified as high risk. These limitations may influence the generalizability and interpretation of our results. Additionally, pharmacological treatments for voiding dysfunction, such as α1-blockers or 5α-reductase inhibitors, were not initiated during the follow-up period. This approach was chosen to avoid introducing potential confounders that could influence the assessment of natural postoperative voiding function and its relationship with bladder cancer recurrence. While this methodological choice allowed for a clearer evaluation of baseline risk factors, we acknowledge that therapeutic interventions may serve a clinically meaningful role in managing residual urine. In particular, future prospective studies should explore whether early intervention in asymptomatic patients with BPH could help reduce the risk of intravesical recurrence, especially in those with elevated post-void residual volumes. Future prospective studies incorporating treatment arms may help clarify the role of such interventions in recurrence prevention.

Nonetheless, our findings provide valuable insights into the potential impact of voiding dysfunction on bladder cancer recurrence. While the results of this prospective study may contribute to more accurate risk stratification and individualized management, further validation is necessary. Ideally, future studies should involve larger, multicenter cohorts and incorporate statistical methods such as propensity score matching to reduce confounding bias.

Prospective investigations that include broader patient populations and extended follow-up periods will be important to clarify the relationship between voiding dysfunction and bladder cancer recurrence. These studies may also help to refine strategies for preventing recurrence in patients with NMIBC.

## Conclusions

In conclusion, our findings suggest that increased residual urine volume in male patients may be associated with a higher risk of intravesical recurrence following TUR-Bt for NMIBC. Addressing voiding dysfunction may therefore represent a meaningful strategy to reduce recurrence risk and improve long-term outcomes in this patient population.
